# Exploring the effects of two-segment loop tunnels on termite food transport efficiency: a simulation study

**DOI:** 10.1093/jisesa/ieae076

**Published:** 2024-07-18

**Authors:** Sang-Hee Lee, Cheol-Min Park

**Affiliations:** Division of Industrial Mathematics, National Institute for Mathematical Sciences, Daejeon, South Korea; Division of Industrial Mathematics, National Institute for Mathematical Sciences, Daejeon, South Korea

**Keywords:** termite, food transport behavior, foraging efficiency, traffic jam, individual-based model

## Abstract

This study explores the food transport efficiency (*E*) in a termite tunnel consisting of a main tunnel and a 2-segment loop tunnel through a model simulation. Simulated termites navigate between the main and loop tunnels through branching nodes (*a*, *b*, *c*, *d*) with associated probabilities (*P*_1_, *P*_2_, *P*_3_, *P*_4_). The loop tunnel locations (*δ*) are considered: near the nest (*δ* = 1), at the center of the main tunnel (*δ* = 2), and close to the food site (*δ* = 3). The results reveal that for *δ* = 1, paths such as *a* → *d* → *b* → *c* and *c* → *d* → *b* → *a* exhibited high *E* values. Conversely, for *δ* = 2, *P*_3_ and *P*_4_ demonstrate elevated *E* values ranging from 0.4 to 0.6. For *δ* = 3, paths like *c* → *d* or *c* → *b* display high *E* values, emphasizing the significance of in-loop separation tunnels (characterized by *P*_3_ and *P*_4_) in alleviating traffic congestion. Partial rank correlation validates that *P*_1_ and *P*_2_ minimally influence *E*, while *P*_3_ and *P*_4_ significantly negatively impact *E*, regardless of *δ*. However, for *δ* = 2, the influence of *P*_3_ and *P*_4_ is notably reduced due to the positional symmetry of the loop tunnel. In the discussion, we address model limitations and propose strategies to overcome them. Additionally, we outline potential experimental validations to ensure a comprehensive understanding of the dynamics governing termite food transport within tunnels.

## Graphical Abstract

**Graphical Abstract:**
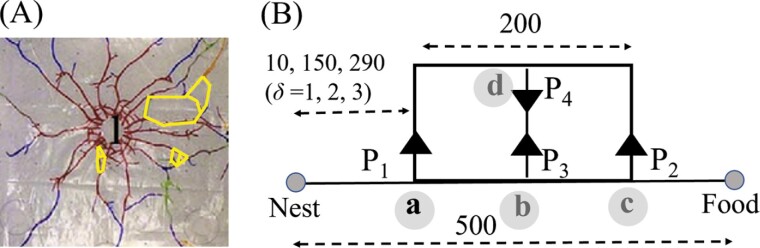

## Introduction

Subterranean termites engage in intricate underground foraging by constructing extensive networks of tunnels that can span tens or even hundreds of meters (King and Spink 1969, Campora and Grace 2001). To maintain colony stability, these termites likely employ behavioral strategies aimed at optimizing both their food search and transport efficiency. Investigating these strategies is crucial for obtaining a comprehensive understanding of termite ecology within tunnels.

Researchers have explored the efficiency of food search by geometrically analyzing tunnel patterns under specific soil conditions. For example, Su and Puche (2003) observed that the area of tunnels in high-moisture sands exceeded those in low-moisture sands. Additionally, Arab and Costa-Leonardo (2005) noted an increase in termite tunneling activity with higher soil temperatures, while Cornelius and Osbrink (2010) found that soil type strongly influenced termite tunneling patterns, with termites avoiding dry sand and clay substrates. Further insights into search efficiency come from Lima and Costa-Leonardo (2012), who demonstrated that termite tunneling is more active in sandy substrates when various physical, chemical, and biological cues are present. Janowiecki and Vargo (2022) reported increased tunneling activity and pattern complexity in *Reticulitermes* species when soldiers were present. However, McCarthy et al. (2023) found, for *Coptotermes formosanus* Shiraki that the presence of soldiers did not impact termite tunneling behavior. Additionally, Bardunias and Su (2010) identified that differences in the balance between excavating the sidewall and waiting for the current tunnel to extend result in distinct tunneling patterns at the tunnel’s end. Collectively, these studies contribute to our understanding of termite search strategies and efficiency under various environmental conditions, unveiling the intricate dynamics within termite tunnel systems.

In addition to the aforementioned experimental studies, researchers have explored simulation studies to further comprehend termite tunnel patterns and food search efficiency. Su et al. (2004) showed that the tunnels of 2 subterranean termite species, *C. formosanus* and *Reticulitermes flavipes* (Kollar), can be described by 10 geometric variables and successfully simulated tunnels based on these variables. Facchini et al. (2020) employed a Laplacian growth model, proposing that termite tunnel patterns result from the combined effects of various parameters.

Despite advancements in understanding food search efficiency, research on food transport efficiency remains limited due to the technical challenge of visually observing termites transporting food particles. To address this challenge, Lee et al. (2022) developed an individual-based model to simulate the food transport process in termites. The authors found that tunnel curvature contributes to increased termite traffic congestion, resulting in reduced food transport efficiency. However, the model faced a limitation in comprehending short tunnels, focusing solely on one highly curved section. To overcome this limitation, Lee and Park (2023) extended an existing model to consider multiple high-curvature tunnel sections and their distributions. They developed a simulation incorporating 4 variables: termite numbers (*k*_1_), distribution of high-curvature sections (*k*_2_), density of such sections (*k*_3_), and time for traffic jam dissipation (*k*_4_). The simulation results mapped transportation efficiency *E*(*k*_1_, *k*_2_), revealing 2 contrasting effects of increasing *k*_1_. More termites led to more traffic jams, decreasing *E*, but also more participants in transportation, increasing *E*.

In this study, we specifically investigated the effect of the 2-segment loop tunnels, frequently observed in real termite tunnel patterns, on food transport efficiency. Michael et al. (2023) demonstrated that termites, when presented with artificial tunnels featuring straight, detour, and detour-twisting structures, tended to create shortcuts between food sources and nests, utilizing these pathways for food transportation. While the experiment simplified the tunnels to 1 of 3 predetermined structures, field conditions often present a more intricate scenario with intertwining tunnels already established. Consequently, as termites forge shortcuts, interactions between tunnels occur, potentially leading to mergers or intersections (Lee et al. 2008). This phenomenon likely results in the formation of 2-segment loop tunnel structures, with the possibility of termites encountering the loop tunnels during food transportation (Campora and Grace 2001, Su and Lee 2023). These loop tunnels have 4 branching nodes, each with its tunnel selection probability. By analyzing selection probabilities, we attempted to understand the ecological mechanisms by which real termites build loop tunnels. In the discussion section, we briefly mention the limitations of our model and propose some ideas to experimentally validate our findings.

## Materials and Methods

### Individual-Based Model

To explore the impact of a 2-segment loop tunnel on termite food transport efficiency, we developed an individual-based model that simulates termite behavior in transporting food particles to the nest. In simplifying the model algorithm, we assumed that all simulated termites are physically identical and engage in the food transport process without utilizing pheromones for signaling. Additionally, we excluded the influences of tunnel wall irregularities and tunnel curvature, both known factors affecting termite behavior in tunnels (Sim and Lee 2013). For the statistical analysis of simulation results, we conducted the simulation 10 times for each combination of control variables and computed the average. Here, the control variables represent probability values dictating which tunnel the simulated termites will choose when encountering a branching node in a tunnel. The duration of each simulation (*T*) was fixed at 5,000 time steps.

### Simulated Termite Tunnel

**Fig. 1.**
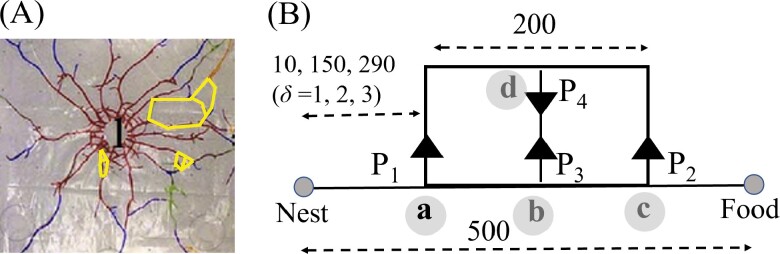
A) Tunnel patterns generated by termites introduced through the labeled introduction hole (“I”) over a 24-h period. The closed lines represent a 2-segment loop tunnel. B) Schematic representation of the tunnel structure, featuring 2-segment loop tunnels and a main tunnel with branching nodes (*a*, *b*, *c*, and *d*). Simulated termites navigate between the main and loop tunnels, guided by probabilities (*P*_1_, *P*_2_, *P*_3_, and *P*_4_) at each node.

Termite tunnel patterns typically consist of a main tunnel and branch tunnels, occasionally featuring a 2-segment loop structure in the branch tunnels, as illustrated in Fig. 1A (for *Coptomermes formosanus*). These loop structures often form in connection with other tunnels rather than independently. The tunnel pattern was formed by 10,000 workers digging for 24 h in homogeneous sand filled between 2 acrylic plates measuring 1.0 × 1.0 m. In our model, we simplified the geometrical structure of 2-segment loop tunnels as a straight main tunnel consisting of 500 grid cells and a loop tunnel with 200 grid cells, connected by branching nodes (refer to Fig. 1B). The main tunnel incorporates 3 branching nodes labeled *a*, *b*, and *c*, while the loop tunnel has 1 branching node labeled *d*. Simulated termites are capable of traversing between the main and loop tunnels through the application of probabilities, specifically *P*_1_, *P*_2_, *P*_3_, and *P*_4_, assigned to nodes *a*, *b*, *c*, and *d*, respectively.

We assume that the separation tunnel, which divides the loop tunnel into 2 sections, is physically shorter in length and wider in width. To reflect this feature in the model, we set a rule that the simulated termites pass through the separating tunnel without spending any time. The model encompasses 3 scenarios: when the 2-segment loop tunnel is in proximity to the nest (*δ* = 1), midway between the nest and the food site (*δ* = 2), and close to the food site (*δ* = 3).

### Food Transport Efficiency

In this model, we defined the termite food transport efficiency (*E*) as the average number of food particles transported to the nest by a simulated termite in a unit time, as follows:


$$\begin{array}{l} E(\tau )=\left(\frac{s\sum_{t=1}^{\tau }food(t)}{\tau N_{0}}\right) \end{array}$$ (1)

Here, *N*_0_ and *τ* denote the number of simulated termites and simulation time devoted to food transportation, respectively. The scaling factor *s*, which was introduced to prevent the value of *E*(*τ*) from becoming too small, was set to 10,000. In this study, the value of *E* is the average value of *E*(*τ*) for 4,500 ≤ *t* ≤ 5,000.

### Behavior of Simulated Termites

**Fig. 2.**
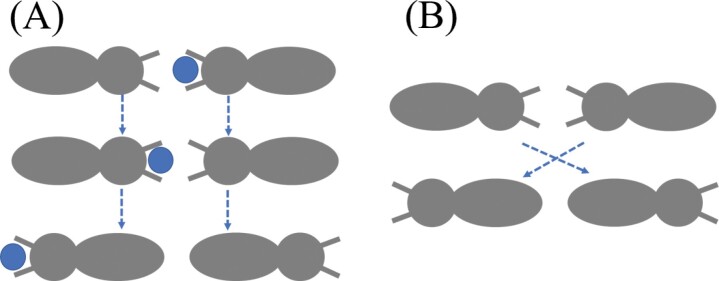
A) A simulated termite carrying food encounters another termite without food. The food-carrying termite transfers food to the empty-handed termite through trophallaxis, a mouth-to-mouth exchange. The food transfer is successful with no loss of food. B) Two simulated termites without food meet in a tunnel. The termites pass each other without stopping or altering their paths.

In this model, when a simulated termite with food encounters another simulated termite without food, it transfers the food with a probability of 0.5 and no food is lost (Fig. 2A). Once the transfer occurs, the 2 simulated termites change direction and continue on their way (Fig. 2B). As a result of this mechanism, when a simulated termite reaches the nest with a food particle, only one food particle is deposited. In instances where no food transfer takes place, simulated termites pass each other without any interaction, mirroring the behavior of real termites navigating through narrow tunnels.

### Traffic Jam

In our model, we set a rule that simulated termites cause a traffic jam when 4 simulated termites converge on the same cell site and the sum of their heading direction vectors is zero. The selection of 4 individuals for this event is based on preliminary studies, which revealed that 6 individuals led to too few traffic jamming events with no observable effect, while employing only 2 individuals resulted in excessive traffic jamming with a lack of food transportation. This careful consideration in choosing the number of termites ensures a balanced simulation, striking the right equilibrium between inducing traffic jams and facilitating the essential transportation of food within the model.

### k-Means Clustering

The *k*-means clustering algorithm, a widely employed unsupervised learning technique, aims to categorize given data into *k* clusters (Sun et al. 2008). This objective is achieved through an iterative process involving 4 steps, all geared toward minimizing the variance of distance differences within each cluster.

Initially, the algorithm arbitrarily sets the initial *k* cluster centroids. Subsequently, it assigns each data point to the nearest cluster centroid. Following this assignment, the algorithm minimizes the variance of the distance between the centroids of each cluster by recalculating the centroid of each cluster. This recalibration is crucial for refining the cluster boundaries. The algorithm iterates through the second and third steps until the cluster centroids no longer undergo any changes. Upon reaching this convergence in step 4, the algorithm effectively groups the data points into distinct clusters. This systematic and efficient approach to clustering in unsupervised learning scenarios ensures a robust methodology for analyzing and categorizing data.

### t-SNE Analysis

The *t-*distributed stochastic neighborhood embedding (*t-*SNE) algorithm, known for its performance in dimensionality reduction and clustering visualization, has been a pivotal tool in various medical fields, including text analysis, image processing, speech recognition, and biochemistry. To achieve the task of visually clustering multidimensional data in 2 dimensions, the algorithm computes 2 conditional probabilities. The first conditional probability, denoted as *p*_*j*|*i*_, represents the likelihood between high-dimensional data, while the second conditional probability, denoted as *q*_*j*|*i*_, signifies the probability between low-dimensional data. These probabilities are precisely defined by Equations (2) and (3) (Hinton and Roweis 2002, van der Matten and Hingon 2008).


$$\begin{array}{l} p_{j|i}=\frac{\exp\left(-{\left|x_{i}-x_{j}\right|}^{2}/2\sigma_{i}^{2}\right)}{\sum_{k\neq i}\exp\left(-{\left|x_{i}-x_{k}\right|}^{2}/2\sigma_{i}^{2}\right)} \end{array}$$ (2)

$$\begin{array}{l} q_{j|i}=\frac{\exp\left(-{\left|y_{i}-y_{j}\right|}^{2}\right)}{\sum_{k\neq i}\exp\left(-{\left|y_{i}-y_{k}\right|}^{2}\right)} \end{array}$$ (3)

Where *x*_*i*_ and *x*_*j*_ are high-dimensional data, *y*_*i*_ and *y*_*j*_ are low-dimensional data, and *σ*_*i*_ is the standard deviation of a normal distribution centered on *x*_*i*_. A natural measure of how well *q*_*j*|*i*_ models *p*_*j*|*i*_ is the Kullback–Leibler divergence (in which case the cross-entropy is equal to an additive constant). SNE uses a gradient descent method to minimize the sum of the Kullback–Leibler divergence over all data points. The cost function *C* is given below.


$$\begin{array}{l} C=\sum_{i}KL(P_{i}|Q_{i})=\sum_{i}\sum_{j}p_{j|i}\log\frac{p_{j|i}}{q_{j|i}} \end{array}$$ (4)

where *P*_*i*_ is the conditional probability of all other targets for a given target *x*_*i*_ and *Q*_*i*_ is the conditional probability distribution of all other targets for a given target *y*_*i*_ (van der Maaten and Hinton 2008). In other words, the more similar the low and high dimensions are, the closer they are to zero. However, the *t-*SNE uses asymmetric conditional probabilities and suffers from the crowding problem, where high-dimensional data that are sufficiently spaced cannot be realized in low dimensions. Therefore, an alternative is to modify the conditional probabilities *p*_*j*|*i*_ and *q*_*j*|*i*_ of the SNE, and the final *t-*SNE can be represented as follows:


$$\begin{array}{l} p_{ij}=\frac{p_{j|i}+p_{i|j}}{2} \end{array}$$ (5)

$$\begin{array}{l} q_{ij}=\frac{1+\exp{\left(-{\left|y_{i}-y_{j}\right|}^{2}\right)}^{-1}}{\sum_{k\neq l}{\left(1+{\left|y_{i}-y_{l}\right|}^{2}\right)}^{-1}} \end{array}$$ (6)

### Partial Rank Correlation Coefficient

To statistically investigate the impact of four variables—*P*_1_, *P*_2_, *P*_3_, and *P*_4_—on food transportation efficiency (*E*), we employed the partial rank correlation coefficient (PRCC) technique, a method for sensitivity analysis (Marino et al. 2008). The PRCC is computed by determining the correlation coefficient (CC) between the input variable *x*_*j*_ and the output variable *y*, as defined by the equation:


$$\begin{array}{l} CC(x_{j},y)=\frac{\sum_{i=1}^{N}\left(x_{ij}-\bar{x}\right)\left(y_{i}-\bar{y}\right)}{\sqrt{\sum_{i=1}^{N}{\left(x_{ij}-\bar{x}\right)}^{2}\sum_{i=1}^{N}{\left(y_{i}-\bar{y}\right)}^{2}}} \end{array}$$ (7)

In this equation, *N* represents the number of samples, and *k* is the size of the input vector. The variable *x* corresponds to 1 of the 4 parameters—*P*_1_, *P*_2_, *P*_3_, or *P*_4_—while *y* represents food transport efficiency (*E*). The strength of the linear relationship between the input variable *x*_*j*_ and the output variable *y* is assessed using the partial correlation coefficient (PCC). PCC eliminates the linear influence of other variables. The PCC is defined as the CC between $x_{j}-{\hat{x}}_{j}\;$ and $\;\;\;y-\;\hat{y}$, where ${\hat{x}}_{j}$ and $\hat{y}\;$ are estimated using the following linear regression model:


$$\begin{array}{l} {\hat{x}}_{j}=c_{0}+\sum_{p=1(\neq j)}^{N}c_{p}x_{p}\;\;\;\;and\;\;\;{\hat{y}}_{j}=b_{0}+\sum_{p=1(\neq j)}^{N}b_{p}x_{p}\; \end{array}$$ (8)

To estimate the regression coefficients *b* and *c*, the study uses the regression fitted variable denoted as “hat”. The PRCC is determined using the linear regression model described in Equation (8), applied to data transformed by ranks ${\hat{x}}_{j}$ and $\hat{y}$. The PRCC ranges from −1 to 1, with positive (negative) values signifying positive (negative) correlations between the parameter and the model’s output. A higher absolute PRCC indicates a more pronounced correlation between the input and output variables. A positive PRCC implies a positive impact on the output *y*, while a negative value suggests a negative contribution to the output.

To streamline the sampling process and minimize redundancy, the study adopts the Latin Hypercube Sampling method, a stratified sampling approach (McKay et al. 1979). This method significantly reduces the number of simulations compared to the Monte Carlo method (Iman and Helton 1988). Specifically, the study conducts 300,000 simulations for 10 × 10 × 10 × 10 × 3 combinations of (*P*_1_, *P*_2_, *P*_3_, *P*_4_, *δ*), each repeated 10 times. Equations (7) and (8) are computed using the “prcc” function available in MATLAB 2021a.

## Results and Discussion

**Fig. 3.**
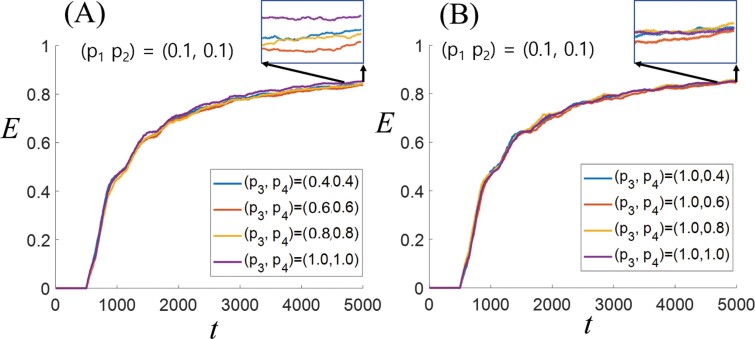
Food transport efficiency (*E*) over time. A) Food transport efficiency (*E*) varies with (*P*_3_, *P*_4_) in the scenario with (*P*_1_, *P*_2_) = (0.1, 0.1), especially at the values of (0.4, 0.4), (0.6, 0.6), (0.8, 0.8), and (1.0, 1.0). B) In the scenario with (*P*_1_, *P*_2_, *P*_3_) = (0.1, 0.1, 1.0), *E* varies when *P*_4_ is set to 0.4, 0.6, 0.8, or 1.0.

To investigate the impact of a 2-segment loop tunnel on food transport efficiency (*E*), we analyzed the variations in *E* with changes in *P*_3_ and *P*_4_ while keeping the values of (*P*_1_, *P*_2_) constant at (0.1, 0.1) (refer to Fig. 3). For *δ* = 2, with (*P*_3_, *P*_4_) values set at (0.4, 0.4), (0.6, 0.6), (0.8, 0.8), and (1.0, 1.0), *E* initially remained at zero for *t <* 500 (Fig. 3A). This early state of *E* = 0 resulted from all simulated termites randomly heading either towards the food site or the nest site without food particles at *t* = 0. The first simulated termite with food took a minimum of 500 timesteps to reach the nest. Subsequently, a sharp increase occurred in the time range of 500 < *t* < 2,000, as simulated termites sequentially picked up and dropped food particles into the nest. The rate of increase then slowed down between 2,000 < *t <* 4,500 and stabilized in the range of 4,500 < *t <* 5,000. Within this convergence range, the combination (*P*_3_, *P*_4_) = (1.0, 1.0) exhibited the highest *E*, while (*P*_3_, *P*_4_) = (0.6, 0.6) showed the lowest. The other 2 cases demonstrated similar *E* values.

To explore scenarios with different *P*_3_ and *P*_4_ values, we set *P*_3_ = 1.0 and observed the variation in *E* as *P*_4_ changed (see Fig. 3B). Overall, *E* followed a similar trend as in Fig. 3A, but the fluctuations in *E* with each *P*_4_ change were relatively small, suggesting that *E* behavior is likely dependent on the combination of *P*_3_ and *P*_4_ values.

**Fig. 4.**
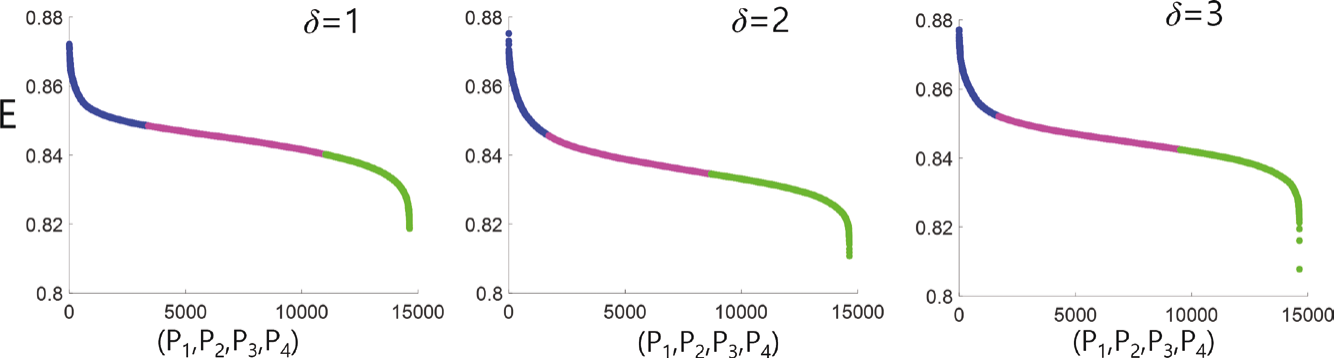
The combinations (*P*_*1*_, *P*_2_, *P*_3_, *P*_4_) for the 3 locations (*δ* = 1, 2, 3) of the loop tunnel are sorted in descending order according to their *E* values, then clustered into 3 groups using *k-*means clustering with *k* = 3.

To investigate the paths with relatively high *E* values in the tunnel, we sorted the combinations of (*P*_1_, *P*_2_, *P*_3_, *P*_4_) in descending order of *E* value for *δ* = 1, 2, 3 (see Fig. 4). Applying *k-*means clustering (*k* = 3) classified them into 3 groups represented by blue (high *E*), pink (medium *E*), and green (low *E*) colors. Regardless of the *δ* value, group 1 showed a sharp downward slope in the *E* distribution, group 2 exhibited a gradual decrease, and group 3 displayed a gradual decrease followed by a sharp decrease. This suggests that simulated termites can achieve a high *E* value by choosing a specific combination of (*P*_1_, *P*_2_, *P*_3_, and *P*_4_).

**Fig. 5.**
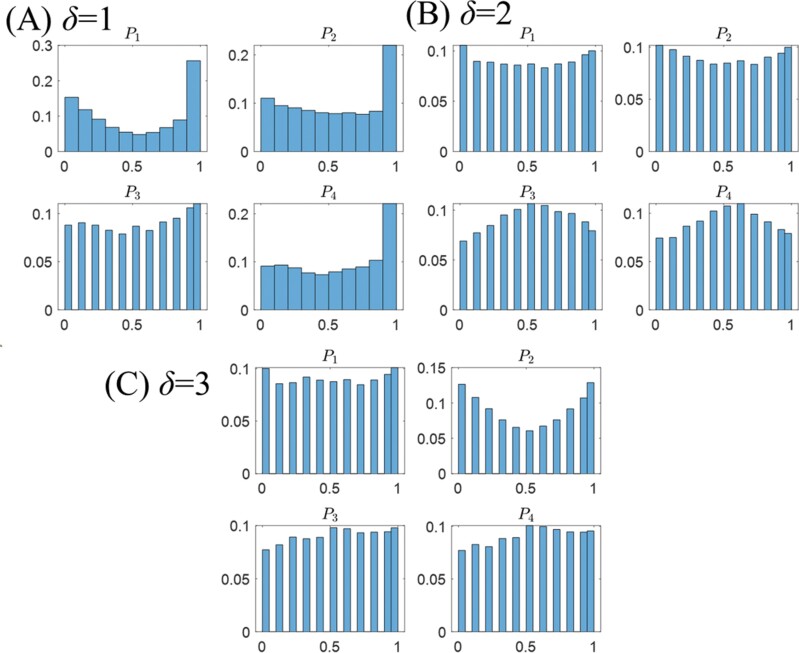
Histograms of *P*_1_, *P*_2_, *P*_3_, and *P*_4_ values for A) *δ* = 1, B) *δ* = 2, and C) *δ* = 3. The *x*-axis shows the intervals, divided into 0.1 intervals within the range of 0 to 1. The *y*-axis shows the frequency of each interval dimension, normalized by the total frequency of all intervals.

To investigate the specific combination, we analyzed the histogram of the values of *P*_1_, *P*_2_, *P*_3_, and *P*_4_ for *δ* = 1, 2, and 3 (see Fig. 5). For *δ* = 1, *P*_1_, *P*_2_, and *P*_4_ showed relatively high frequencies between 0.9 and 1.0, while *P*_3_ exhibited an uncharacteristic distribution. This suggests that it is advantageous for simulated termites to use the *a* → *d* → *b* → *c* (nest to food site) and *c* → *d* → *b* → *a* (food site to nest) pathways to obtain high *E* values. In other words, termites have to take these routed instead of a direct *c* → *b* → *a* because of congestion. On the other hand, for *δ* = 2, *P*_1_ and *P*_2_ showed almost uniform distributions, while *P*_3_ and *P*_4_ had distributions that increased from 0.5 to 0.6. This indicates that it is advantageous for simulated termites to take the *d* → b or *b* → *d* paths but with a probability that is not too high, to improve their *E*. For *δ* = 3, *P*_1_, *P*_3_, and *P*_4_ exhibited roughly uniform values, with only *P*_2_ showing high frequencies at 0.0–0.1 and 0.9–1.0. This means that when the simulated termites return to the nest from the food site, their taking the path *c* → *d* or *c* → *b* has a positive effect on improving the *E* value.

**Fig. 6.**
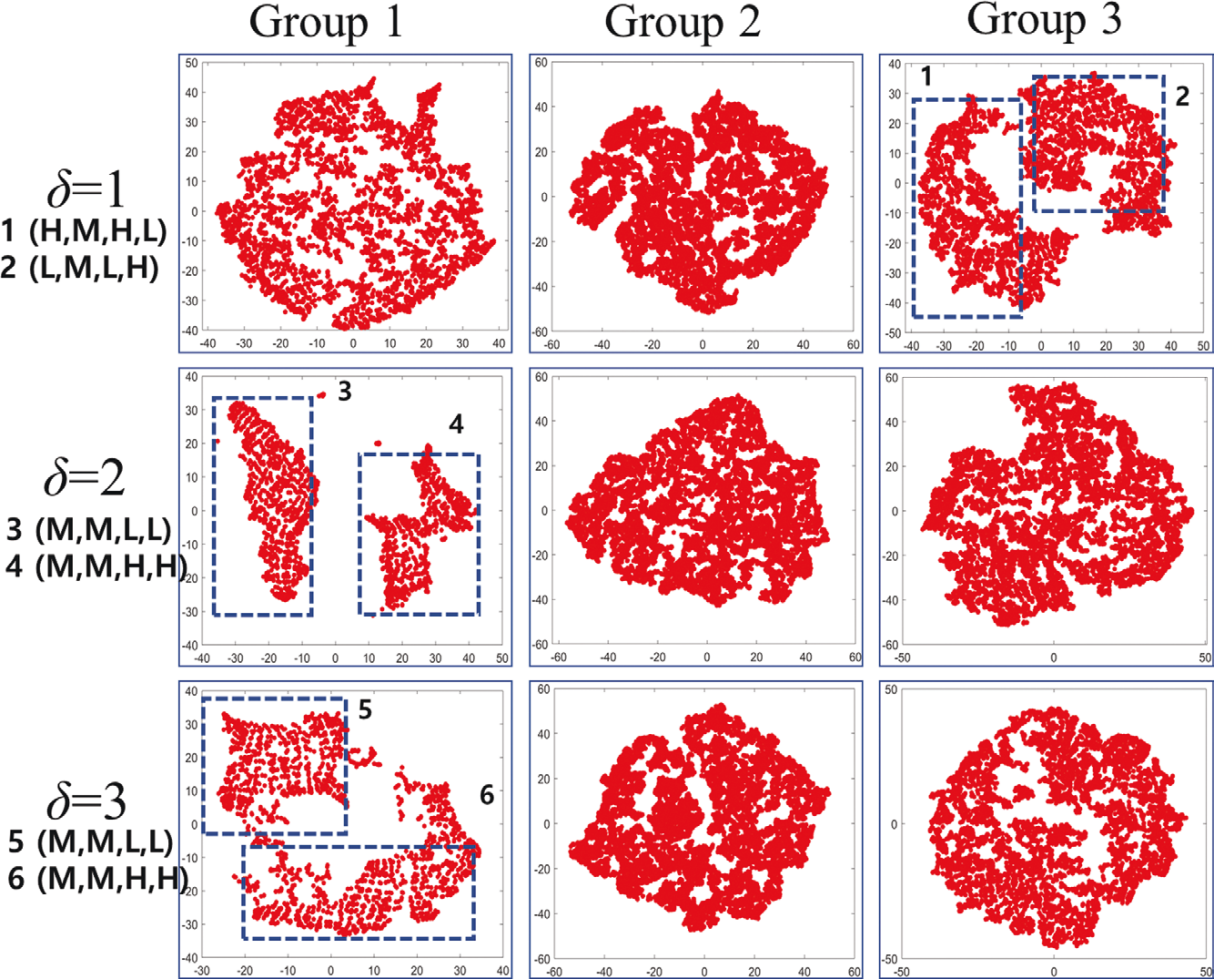
*t-*SNE analysis for various (*P*_1_, *P*_2_, *P*_3_, *P*_4_) combinations across *δ* = 1, 2, and 3 reveals distinct clusters. Dotted rectangles highlight with prominent clustering. Within each region, mean values of *P*_*1*_, *P*_2_, *P*_3_, and *P*_4_ fall into 3 categories: 0.0–0.33 (Low: *L*), 0.33–0.66 (Medium: *M*), and 0.66–1.0 (High: *H*).

**Fig. 7.**
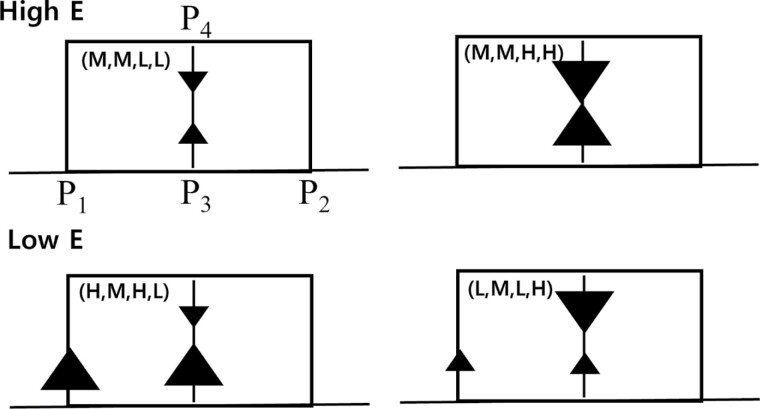
Illustration of paths with high *E* (upper) or low *E* (lower) for the combinations of (*P*_1_, *P*_2_, *P*_3_, *P*_4_).

The aforementioned paths were deduced solely based on the distributions of *P*_1_, *P*_2_, *P*_3_, and *P*_4_, without considering any information about the node order. This means that alternative paths leading to high *E* values are likely to exist. To address this, we conducted a *t*-SNE analysis of *E* values for path combinations (*P*_1_, *P*_2_, *P*_3_, and *P*_4_) belonging to groups 1, 2, and 3 for *δ* = 1, 2, and 3 (Fig. 6). For *δ* = 1, groups 1 and 2 did not exhibit a distinct clustering tendency, whereas group 3 was clearly separated into 2 clusters (cluster 1 and cluster 2). This suggests the presence of other paths with high *E* within group 1, beyond those mentioned in Fig. 5A. For *δ* = 2, group 1 divided into 2 clusters (cluster 3 and cluster 4), and for *δ* = 3, group 1 split into cluster 5 and cluster 6. To identify the paths within each cluster, we categorized the values of *P*_1_, *P*_2_, *P*_3_, and *P*_4_ into “*H”* (0.66–1.0), “*M”* (0.33–0.66), and “*L”* (0.0–0.33), visually simplifying the paths (Fig. 7). Paths with high *E* values were found when simulated termites exhibited a relatively high or low probability of selecting the separation tunnel, characterized by nodes *b* to *d*. This figure illustrates that changes in *P*_2_ and *P*_4_, determining the characteristics of the separation tunnel, exert a significant influence on *E*. It is important to note, as discussed in Section 2.2, that simulated termites encounter no time delay when traveling between the main and loop tunnels. From this, we can infer that the variations in *E* are linked to the traffic conditions of the simulated termites rather than the separation tunnel itself.

**Fig. 8.**
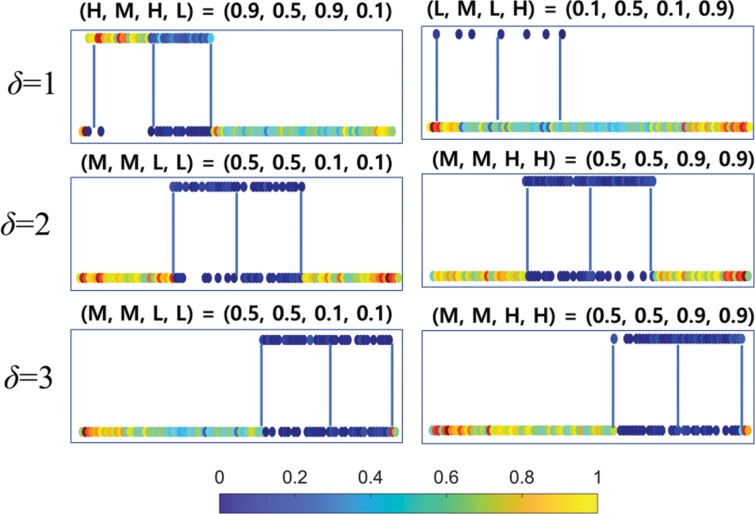
The top 2 figures show examples of low-efficiency (*E*) paths in *δ* = 1, with the combinations (*P*_1_, *P*_2_, *P*_3_, *P*_4_) = (*H*, *M*, *H*, *L*) and (*L*, *M*, *L*, *H*). The middle and bottom figures show examples of high-efficiency (*E*) paths in *δ* = 2 and 3, with the combinations (*P*_1_, *P*_2_, *P*_3_, *P*_4_) = (*M*, *M*, *L*, *L*) and (*M*, *M*, *H*, *H*), respectively. The color of the circles represents the normalized frequency of traffic jams that occur at the corresponding site.

To validate this inference, we examined the traffic jam ratio for different path combinations (Fig. 8). Circles on the graph represent locations where traffic jams occurred, with the color indicating the ratio value. For *δ* = 1 and certain combinations (e.g., (*H*, *M*, *H*, *L*) and (*L*, *M*, *L*, *H*)), high traffic congestion was observed on the *a* → *d* (or *d* → *a*) path of the 2-segment loop tunnel and the *a* → *c* or (*c* → *a*) path on the main tunnel. Conversely, for *δ* = 2 and 3, combinations such as (*M*, *M*, *L*, *L*) and (*M*, *M*, *H*, *H*) showed significantly lower traffic congestion on both *a* → *c* and *c* → *a* paths. This observation confirms that the separation tunnel impacts *E* by properly distributing simulated termites in the main and loop tunnels, effectively reducing traffic jam frequency.

**Fig. 9.**
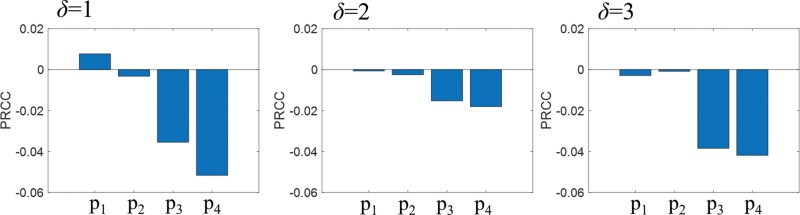
Analysis of partial rank correlation coefficient (PRCC) values for the 4 control variables, *P*_1_, *P*_2_, *P*_3_, and *P*_4_, demonstrating the positive or negative impact of each variable.

For a comprehensive understanding of the impact of *P*_1_, *P*_2_, *P*_3_, and *P*_4_ on *E*, we conducted a PRCC analysis (Fig. 9). The results indicate that *P*_1_ and *P*_2_ exert a relatively minor influence on *E*, irrespective of the value of *δ*. In contrast, *P*_3_ and *P*_4_ have a substantial impact on *E*. Specifically, at *δ* = 2, the influence of *P*_3_ and *P*_4_ is somewhat reduced compared to that of *δ* = 1 and 3, primarily because the loop tunnel is positioned at the center of the main tunnel.

## Additional Discussion

The primary objective of this study is to investigate the efficiency of food transport within termite tunnel networks, particularly those featuring a main tunnel and a 2-segment loop tunnel, a configuration frequently found in nature. The loop tunnels arise from the convergence of main and branch tunnels as the termites excavate their intricate network, and they may also emerge as efficient shortcuts created by the termites while transporting food resources to the nest.

**Fig. 10.**
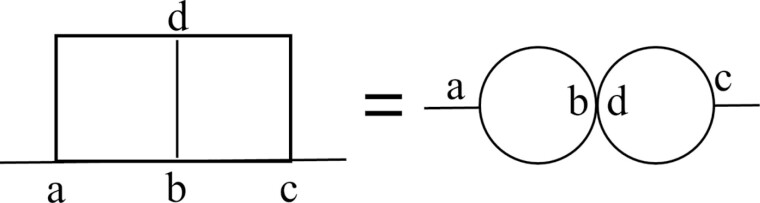
A topological structure characterized by identical features of the 2-segment loop tunnels.

The simulation results unveiled a crucial connection between transport efficiency and the presence of a separation tunnel, which divides the loop tunnel into 2 distinct segments. This separation tunnel efficiently dispersed simulated termites throughout both the main and loop tunnels, mitigating population density in specific areas and minimizing traffic congestion. The critical role of the separation tunnel arises from its topological equivalence to 2 interconnected single-segment loop tunnels that share a single contact point, as illustrated in Fig. 10. To facilitate understanding, let us consider a scenario where simulated termites strategically choose paths such as *a → d → b → c* (or *a → b → d → c*) when heading to a food site, and *c → d → b → a* (or *c → b → d → a*) when heading to the nest. The contact points enable simulated termites to move seamlessly between the main and loop tunnels without encountering time delays or traffic jams. Physically characterizing this contact point with a short length and a wide width is crucial, which allows termites to pass through quickly, resulting in shorter transit times and less traffic congestion due to reduced interactions between individuals (Michael et al. 2023). The characteristics of this contact point emphasize the importance of considering both spatial and topological factors when aiming to comprehend the efficiency of food transportation in termites.

Naturally, a pertinent question arises: how do real termites make decisions to select paths that optimize transport efficiency? Lee et al. (2016) provide a compelling answer: Termites employ their antennae at branching nodes to gather information regarding the width and angle of branching tunnels. Among the main and branch tunnels, they exhibit a preference for the wider tunnel more frequently, while the angle serves to reinforce their selection.

An experimental test of this hypothesis could involve a comparison of the widths of the main and branch tunnels while simultaneously measuring branching angles. For instance, if termites aim to enhance the value of *P*_1_, they can achieve this by increasing the width of the branching tunnels adjacent to node a during their tunneling activity and ensuring that the branching angle is smaller than the typical 45° observed in standard tunnel patterns (Lee et al. 2016).

However, it is crucial to interpret this hypothesis within the context of its simplified model, designed for algorithmic efficiency. The model overlooks certain aspects of real termite tunnels, such as tunnel curvature and surface irregularities. Additionally, it assumes a centralized location for the separation tunnel, which may not always align with reality, as observed loop tunnels often feature the separation tunnel offset to the left or right. Furthermore, the model treats termite individuals as identical particles, neglecting potential biological variations in walking activity and carrying capacity (Arab and Costa-Leonardo 2005, Lima and Costa-Leonardo 2012).

Despite these limitations, this study holds significance as a pioneering exploration of termite food transport efficiency, an aspect not well studied due to the inherent difficulty of observation. Moreover, it provides valuable insights that can guide future experiments aimed at validating the results obtained through simulation.
